# The design and implementation of an obstetric triage system for unscheduled pregnancy related attendances: a mixed methods evaluation

**DOI:** 10.1186/s12884-017-1503-5

**Published:** 2017-09-18

**Authors:** Sara Kenyon, Alistair Hewison, Sophie-Anna Dann, Jolene Easterbrook, Catherine Hamilton-Giachritsis, April Beckmann, Nina Johns

**Affiliations:** 1 Institute of Applied Health Research, College of Medical and Dental Sciences, University of Birmingham, Edgbaston, Birmingham, B15 2TT UK; 2 Institute of Clinical Sciences, College of Medical and Dental Sciences, University of Birmingham, Edgbaston, Birmingham, B15 2TT UK; 3 Day Assessment Unit, Birmingham Women’s and Children’s NHS Foundation Trust, Mindelsohn Way, Birmingham, B15 2TT UK; 4 University of Bath, Claverton Down, Bath, BA2 7AY UK; 5 Pacific Institution, 33344 King Road, Abbotsford, BC V2S 4P4 Canada; 6 Birmingham Women’s and Children’s NHS Foundation Trust, Mindelsohn Way, Birmingham, B15 2TG UK

**Keywords:** Obstetric triage system, standardised assessment, priority 6 words

## Abstract

**Background:**

No standardised system of triage exists in Maternity Care and local audit identified this to be problematic. We designed, implemented and evaluated an Obstetric Triage System in a large UK maternity unit. This includes a standard clinical triage assessment by a midwife, within 15 min of attendance, leading to assignment to a category of clinical urgency (on a 4-category scale). This guides timing of subsequent standardised immediate care for the eight most common reasons for attendance. A training programme was integral to the introduction.

**Methods:**

A mixed methods evaluation was conducted. A structured audit of 994 sets of maternity notes before and after implementation identified the number of women seen within 15 min of attendance. Secondary measures reviewed included time to subsequent care and attendance. An inter-operator reliability study using scenarios was completed by midwives. A focus group and two questionnaire studies were undertaken to explore midwives’ views of the system and to evaluate the training. In addition a national postal survey of practice in UK maternity units was undertaken in 2015.

**Results:**

The structured audit of 974/992 (98%) of notes demonstrated an increase in the number of women seen within 15 min of attendance from 39% before implementation to 54% afterwards (RR (95% CI) 1.4 (1.2, 1.7) *p* = <0.0001).

Excellent inter-operator reliability (ICC 0.961 (95% CI 0.91–0.99)) was demonstrated with breakdown showing consistently good rates.

Thematic analysis of focus group data (*n* = 12) informed the development of the questionnaire which was sent to all appropriate midwives. The response rate was 53/79 (67%) and the midwives reported that the new system helped them manage the department and improved safety.

The National Survey (response rate 85/135 [63%]) demonstrated wide variation in where women are seen and staffing models in place. The majority of units 69/85 (81%) did not use a triage system based on clinical assessment to prioritise care.

**Conclusions:**

This obstetric triage system has excellent inter- operator reliability and appears to be a reliable way of assessing the clinical priority of women as well as improving organisation of the department. Our survey has demonstrated the widespread need for implementation of such a system.

**Electronic supplementary material:**

The online version of this article (10.1186/s12884-017-1503-5) contains supplementary material, which is available to authorized users.

## Background

Triage systems ensure that patients receive the level of care appropriate to their clinical priority, and that resources are used effectively. Such systems are common in Emergency Medicine Departments with many based on the Manchester Triage System (MTS), launched in the UK in 1997 [1]. The MTS is designed to standardise assessment and increase reproducibility and validity [2, 3] and has been mandated for use in UK Accident and Emergency Departments since 2002.

In maternity care, triage of pregnant women is less reliable [4, 5], and the need to develop specific guidelines and education packages [6] has been identified, with limited evidence of such a system being implemented and evaluated in the UK [7, 8]. Failure to identify and treat women with unscheduled pregnancy-related attendances has resulted in adverse outcomes [9, 10].

The physiological changes associated with pregnancy mean the general parameters of standard triage tools may not be applicable, as pregnancy is associated with an increased resting heart rate, lower blood pressure and increased respiratory rate in the mother. This together with the underlying good health of the maternity population may mask the severity of maternal illness unless a specific assessment is undertaken by appropriately trained health care professionals. There is also no means of assessing the condition of the unborn baby in existing triage tools.

The maternity unit which was the main site of this study has approximately 8000 births annually and some 1200 women attend the Triage Department each month. An initial audit of the triage department identified delays in the assessment and treatment of women, and variation in the observations and investigations undertaken during the initial assessment and subsequent care of the women. Women would wait to be seen in the order in which they attended, and while informal triaging would be undertaken for those in obvious need of urgent attention, the remaining women would wait for varying amounts of time depending on the work load and staffing levels. Women stayed in the same room and were cared for by the same midwife (one to one care) throughout the care episode, which potentially led to ‘blocking’ of rooms by women with low levels of clinical urgency.

The midwives had expressed concerns about patient safety and workload and there was commitment in the team to change how the Department functioned.

This paper reports the development, implementation and initial evaluation of a standard Birmingham Symptom specific Obstetric Triage System (BSOTS) for the assessment of women attending a large maternity unit for unscheduled pregnancy related reasons. The BSOTS was designed to improve safety and standardise care.

### The Birmingham symptom specific obstetric triage system: Development and implementation

The system was co-produced by researchers and clinicians led by an obstetrician and a researcher, and involved a group of senior midwives working on Delivery Suite and in Triage who formed a Development Group (DG). Co production in this context being a process involving clinicians and researchers working alongside each other at almost all stages of the project [11]. An Advisory group was also convened to oversee the development and implementation of the system.

The key clinical indicators and their parameters (guided by those used by the MTS [12]) for the initial assessment (triage) defined the level of clinical urgency using a 4-category scale. The guidelines for immediate care and investigation were developed by the DG using the available evidence, and consensus statements with the agreement of the local obstetric consultants.

**Fig. 1:**
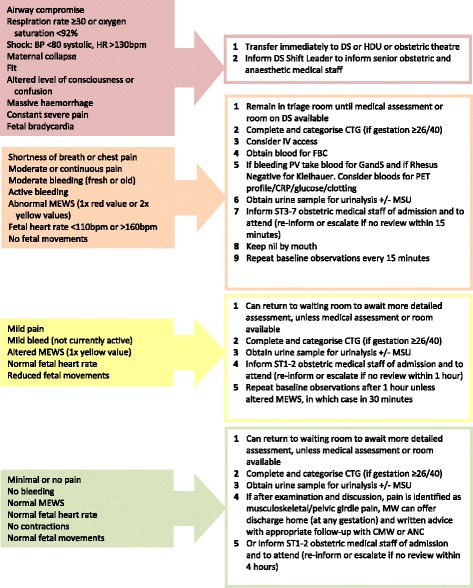
Example of clinical discriminators and level of urgency assigned for Antenatal bleeding

The BSOTS included:

- Completion of a standard clinical triage assessment by a midwife within 15 min of a woman’s attendance. This includes taking a brief maternal history, completion of baseline maternal observations (temperature, pulse, respirations and blood pressure), assessment of pain levels (using a numerical pain score), abdominal palpation and auscultation of the fetal heart rate (if the woman was antenatal).
- This assessment is used to define a category of clinical urgency, which guides timing of subsequent assessment and immediate care (by an obstetrician if required).
- Categories of clinical urgency (see Fig. 1 for example) were defined as:
  - ◦ Red: immediate further assessment
  - ◦ Orange: further assessment within 15 min
  - ◦ Yellow: further assessment within an hour
  - ◦ Green: further assessment within 4 h
- Standardised symptom-specific algorithms were developed for allocation of clinical priority and the immediate care and further investigation of the eight commonest reasons for attendance (abdominal pain, antenatal bleeding, hypertension, suspected labour, ruptured membranes, reduced fetal movements, unwell/other, and postnatal concerns). Documentation is provided to support and standardise completion of the clinical tasks required (see example in Fig. 1).
- The introduction of the BSOTS was supported by a comprehensive staff training programme.

#### Changes to work practices

In order to increase the ‘flow’ of women through the department (identified as being problematic) women assessed as being of low clinical urgency following the triage (i.e., those allocated Yellow or Green) would return to the waiting area until their next assessment was due, or the medical staff were available. The intention was that women with higher levels of clinical urgency (red and orange) would be identified within 15 min of arrival and reviewed sooner by senior medical staff or transferred immediately to delivery suite or the High Dependency unit on delivery suite.

The introduction of the system also meant that one midwife would undertake the initial triage assessment and a second midwife would complete the subsequent assessments and co-ordination of care. Standardising clinical assessments and subsequent care was also a new approach (see Additional file 1: Figure. S1).

#### Training of staff

The triage unit is staffed by two midwives from the staff complement of the Delivery Suite, so potentially in excess of 120 midwives could be assigned to work in the area. Prior to implementation, an interactive training package was developed and delivered to 70 midwives (who were likely to work there) over an 8 week period, focusing mainly on those who were regularly assigned to triage. The training was based on the Australian Emergency Triage Education Kit [13] and lasted a maximum of 3 h. Content included an introduction to Triage (purpose of triage, history, timescales and the role of the Triage midwife), communication and assessment of pain, the new BSOTS system and the changes to working practices required for it to be implemented. Scenarios for each of the symptoms and levels of urgency were included and used to simulate the use of the BSOTS by the participants. A similar, shorter training session was provided for medical practitioners as their needs were different. The BSOTS was implemented in April 2013. Additional support and training were provided by the development group for the first few weeks following implementation.

## Methods

A mixed methods design was selected as the best approach for evaluating the impact of the introduction of the BSOTS. Quantitative and qualitative methods were used in a balanced way to access the key aspects of the phenomenon being investigated [14]. This involved:

- A structured audit of notes for a set period before and after implementation
- An inter-operator reliability study of the triage tool, using scenarios completed by clinical midwives
- Exploration of midwives’ views using:
  - ◦ Focus groups
  - ◦ A questionnaire to investigate midwives’ views of implementation
  - ◦ Questionnaires to assess the bespoke training immediately after training and at 3 months
- National survey designed after implementation to explore UK clinical practice for the assessment and management of unscheduled pregnancy related attendances

### Structured audit of notes

#### Primary measure:

- Number of women having initial triage assessment within 15 min of arrival

#### Secondary measures (classified by level of urgency)

- Time to midwifery assessment subsequent to arrival
- Time to medical assessment, if required
- Total time spent in Triage department
- Whether the woman was admitted (and to where) or discharged and by whom
- Date of next contact and reason for attendance, if discharged
- Reliability and validity of the assigned category of urgency following the initial triage assessment was undertaken by reviewing the notes of women/babies who had predefined outcomes within 24 h of attendance (these included maternal admission to High Dependency Unit/Intensive Therapy Unit or death, category 1 Caesarean Section, active neonatal resuscitation, Apgar <7 at 5 min, arterial pH <7.05 or neonatal admission to Neonatal Intensive Care Unit or neonatal death)

#### Sample size

The Development Group estimated that 60% of women had an assessment within 15 min of attendance in Triage before the introduction of BSOTS. It was suggested this would increase to 70% following the introduction of BSOTS. To detect a difference of this size (10% absolute) with 90% power (5% significance) would require at least 992 notes to be audited (496 before introduction and 496 afterwards).

We selected a random sample of 992 from the notes of the 1074 women who attended in June 2012 and 1028 in June 2013. The audit was undertaken by members of the DG following a pilot on 20 sets of maternity notes (from July 2012), undertaken to refine the data collection form. Data were extracted using an audit data collection form. For the year before implementation (2012) data was extracted from the medical notes and for the year of implementation (2013) data was extracted from the triage specific documentation. In order to account for any possible differences in the data extraction that may have occurred between the two time periods (June 2012 and June 2013), the notes were reviewed in batches of 50 from each year.

For the proportion of women assessed within 15 min in 2013 compared with 2012, relative risks and 95% confidence intervals were generated; statistical significance was determined through chi-squared test. Two sample t-tests were used to evaluate whether there was evidence of a difference from 2013 compared with 2012 in time waiting for initial assessment, between arrival and medical assessment and total time in triage. Descriptive statistics are used for the remaining data.

### Inter-rater reliability study

Vignettes were devised to characterise the eight primary reasons for attendance and the clinical observations (determinants) that are relevant to the decision making. The scenarios included women presenting with symptoms consistent with each of the four classifications (i.e., red, orange, yellow or green) and each of the reasons for attendance. Thirty clinical midwives were presented with the scenarios one at a time and asked to assign each case to a triage category using the eight BSOTS algorithms available in the practice setting, to guide their decision making. Basic demographic data regarding the midwives’ seniority and number of years working in maternity care was recorded.

A exploration was undertaken [15] to establish the required sample size using eight vignettes. For intraclass correlations, two-tailed, alpha of 0.05 and power of 0.8: 4 raters would be required to detect a large effect size (*r* = 0.5), 8 raters for a medium effect size (*r* = 0.3) and 30 for a small effect size (*r* = 0.1). Thus, given the timelines and number of midwives using the system we conducted the study to detect a small effect (i.e., 30 raters). This also allowed for comparison between the different levels of clinical midwives (15 senior and 15 less senior midwives) with each group completing the eight scenarios.

To ensure comprehensive examination of the inter-relator reliability (IRR) of the BSOTS, both weighted [calculated as an Intraclass Correlation [16, 17] (ICC)] and non-weighted IRR were calculated [18, 19] as well as rates of accuracy [20].

### Exploration of midwives’ views

To investigate midwives’ views and experiences of the implementation of the BSOTS two focus group interviews [21] were held with midwives (12 in total). Thematic analysis [22] of the focus group data was undertaken to inform the development of a questionnaire. The questionnaire was designed to explore midwives’ views immediately after training and again 3 months later. The resulting data were analysed using descriptive statistics.

### National Survey of practice

A national paper based postal survey was distributed to Labour Ward Leads in UK maternity units with over 3000 births (135), to explore where women with unscheduled pregnancy related attendances were seen within their maternity units, the times such services were available, and the midwifery and obstetric staffing models used. Enquiry was also made as to whether the unit currently used a system to identify clinical priority of those attending, and what that system was.

## Results

### Structured audit of notes

Data was extracted from 974/992 (98%) sets of maternity notes as 18 sets were not available. Baseline characteristics were similar for all those who attended the Triage department and those included in the audit (for parity, maternal age and ethnicity- Additonal file 2: Table S1) and between the 2 years audited (for parity, maternal age and ethnicity, primary reason for attendance and attendance number- Additional file 2: Table S2). In 2013, the commonest reason for attendance was suspected labour (23%), with reduced fetal movements accounting for a further 19%. Hypertension (2%) was the least common reason.

The primary measure of the introduction of the BSOTS (Table 1) was an increase in the number of women seen within 15 min of attendance from 39 to 54%. Relative risk (RR) 1.4 (1.2, 1.7 (95% confidence interval (CI) . More women were assigned to the orange, yellow and green categories of urgency and there was a reduction in the time taken for initial assessment, when compared with the results of the audit undertaken before the system was introduced. The number of women assigned to the red category was low, as they normally bypassed triage and were seen directly on Delivery Suite immediately following admission (Additional file 2: Table S3).

**Table 1:** Primary measure – Proportion of women assessed within 15 min of attendance^a^

	2012 *n* = 496		2013 n = 496		Relative risk (95% CI); *p*-value
Seen within 15 min	159/421	38%	209/391	53%	1.4 (1.2,1.7); p = <0.0001
Time of first assessment not available	75^b^		105^c^		

^a^For women who attended in 2012 this was the time from arrival to midwifery assessment and for women who attended in 2013 this was the time of arrival to time of initial triage. Women categorised as ‘Red’ were taken straight to Delivery Suite and not seen in Triage

^b^The time was not recorded for 75 women in 2012 (8 were not assessed in Triage, the time of assessment was not recorded for 57 women, and 10 sets of notes were not available)

^c^The time was not recorded for 105 women in 2013 (31 were not assessed in triage, the time of assessment was not recorded for 66 women, and 8 sets of notes were not available)

*Secondary outcomes:* Introduction of BSOTS appears to result in reductions in the time spent waiting for assessment and the time to medical assessment, as well as reductions in the total time spent in triage- Table 2.

**Table 2:** Comparison of the time of initial assessment, medical assessment and total time in triage in 2012 and 2013

Waiting times	Orange		Yellow		Green	
	2012	2013	2012	2013	2012	2013
Time waiting for initial assessment^a^	*n* = 102	*n* = 71	*n* = 200	*n* = 136	*n* = 104	*n* = 66
Waiting time – median	00:28	00:14	00:22	00:16	00:28	00:16
Standard Deviation	00:37	00:30	00:54	00:25	00:40	00:32
*p* value	0.008		<0.001		0.012	
Time between arrival and medical assessment	*n* = 68	*n* = 39	*n* = 92	*n* = 84	*n* = 47	*n* = 42
Waiting time – median	01:29	01:16	01:09	01:00	01:13	01:00
Standard Deviation	00:45	01:50	01:07	01:39	00:45	00:50
p value	<0.001		<0.001		0.655	
Total time in triage^b^	*n* = 87	n = 49	*n* = 151	*n* = 86	*n* = 75	n = 49
Waiting time – median	02:03	01:32	01:38	01:58	01:58	01:55
Standard Deviation	01:34	01:14	01:11	01:32	01:03	01:19
p value	<0.001		0.228		0.354	

^a^In 2012 this is the time between arrival and midwifery assessment, in 2013 this is the time between arrival and triage assessment

^b^Across both years this is the time between arrival and discharge/admission to another location

N.B. This table only contains women for whom the relevant times were recorded

Seventy six per cent of women were discharged home following assessment (data not shown). Of those admitted, the most common reason was suspected labour. The date and venue of next attendance were collected to determine the reliability of the triage assessment, and can be found in Additional file 2: Table S4. The majority of women were seen over a week later (60%), mainly for scheduled antenatal visits, 32% of women gave birth and date of next contact is unknown for 7%.

Review of the 27 cases with serious maternal or neonatal events (defined above) within 24 h of Triage assessment provided evidence of the reliability and validity of the standardised triage assessment, with these events occurring a number of hours after the triage assessment and being related to labour/ birth.

### Inter-rater reliability study

Thirty clinical midwives, who had undergone the BSOTS training and were currently working in Triage were randomly selected and agreed to participate in the study.

The lower level clinical midwives were more likely to be rotational than more senior staff, have worked for a shorter time in midwifery, and be younger. There were no other differences between band levels and demographics (Additional file 2: Table S5). Midwives most frequently worked in triage 1–2/week (43.3%, followed by 1–2/month, 36.7%), and rated the tool as extremely (43.3%) or fairly useful (50.0%).

Excellent inter-rater reliability (ICC 0.961 (95% CI 0.91–0.99) was demonstrated [23] (Table 3). Total accuracy was 90.8%, and scenarios with the highest and lowest clinical importance were most consistently assessed accurately. Chi square goodness-of-fit calculation indicated the frequency of incorrect answers for the yellow category was significantly higher than in all other categories [χ^2^ (2) = 27.91, *p* < .001].

**Table 3:** Accuracy and inter-rater reliability

Measure	Percentage of agreement	ICC (95% CI)	Unweighted IRR (95% CI) ^c^
Green	98.33%	a	0.94 (0.93–1.0)
Yellow	68.33%	0.50 (0.15–0.99)^b^	0.70 (0.67–0.85)
Orange	96.67%	a	0.83 (0.82–0.88)
Red	100%	a	0.97 (0.96–1.00)
Overall	90.83%	0.961 (0.91–0.99)	0.85 (.85–.89)

^a^unable to compute ICC due to low variance – scores are to highly consistent

^b^Number should be interpreted with caution due to low variance and large CIs

^c^Light Kappa statistic used to compute unweighted IRR

^d^Bootstrap – t utilised to adjust confidence intervals [1]

There was no apparent relationship between demographic variables (e.g., band level, triage experience), and the level of accuracy or inter-rater agreement (*p* > 0.05). The ‘yellow’ scenario for reduced fetal movements accounted for 68.2% (*n* = 15) of all incorrect answers which were consistently an upgrading of risk from yellow to orange risk level. Upgrading of priority (*n* = 17; 77.3% of incorrect responses) was more common than downgrading (*n* = 5; 22.7% of incorrect responses).

### Focus groups and questionnaire 1 to assess midwives’ views of implementation

Findings from the focus group interviews demonstrated that the midwives felt the introduction of the new system had gone well and that it helped them manage and organise the department. They reported that they felt the safety of women and their babies had improved and that the system, although standardised, afforded them opportunities to use their clinical judgment when appropriate. However, both the focus group participants and the questionnaire respondents identified unnecessary repetition in the paperwork and expressed concerns about the use of a validated numerical pain score, which was felt to be a limitation of the system.

The response rate to the questionnaire was 53/79 (67%) and data was analysed using descriptive statistics (Additional file 3). All the midwives who responded to the questionnaire worked in Triage often and the majority had undertaken the training (38/53). The findings suggest that the midwives found the system ‘largely helpful’ or ‘extremely helpful’ in assessing the clinical urgency of women attending (37/53), that it ‘usually’ or ‘always’ allowed them to use their clinical judgement (38/53), despite being standardised, and ‘usually’ or ‘always’ enabled them to accurately describe the workload in the Triage department (35/53). Some midwives found the lack of continuity when not caring for individual women (a feature of the previous system) difficult to adjust to, and, 36/53 midwives felt it was safer to divide care into immediate clinical assessment and further care and investigations. The responses regarding the pain score showed that midwives’ opinions were divided, with some feeling that it should be agreed between the midwife and the women, and others stating that only midwives should assess pain.

### Questionnaire 2-to assess midwives’ views of the bespoke training

The response rate immediately after training was 100%, and 69% (49/65) at 3 months. Four midwives were unavailable for follow-up at 3 months (Additional files 4 and 5).

Responses showed that the midwives felt the training had improved both their knowledge of and confidence in using the new system on completion and 3 months after implementation. Three months after training, the midwives who responded to the questionnaire (49/65) gave some insights into how the system was working which were similar to the questionnaire responses described above. Forty three out of forty nine commented positively, and some staff felt the system helped them prioritise care (13) and enabled them to assess the women’s needs more quickly. When asked what needed improving, 44 midwives commented; with 10 stating that the pain score needed to be reviewed, 7 saying the availability of doctors was an issue and 6 feeling the paperwork was repetitive.

### National Survey of practice

The postal survey was undertaken in 2015 and had a response rate of 63%- 85 of 135 maternity units. Most women with unscheduled pregnancy related attendances were seen in units designated as either Triage or Day Assessment 69/85 (81%), with 61/85 (72%) reported to be open 24 h a day. Nineteen percent of the respondents (16/85) reported that women were seen on Delivery Suite. The number of women being seen monthly varied from less than 300 (3 units) to over 1000 (17 units) and reflects the size of the respective maternity units that responded. Fifty three reported separate staffing from Delivery Suite and 18 reported shared staffing. Models of staffing reflected the variation in size of the units and services provided. For example there were differences in the amount of cover, the seniority and number of midwives and obstetricians available, and 4 units employed maternity support workers. Thirty four units reported not having a system in place to identify the clinical priority of women presenting with unscheduled pregnancy related problems. Of the 48 units that did report using such a system, 35 reported it was based solely on clinical judgement. In summary, 69 of the 85 (81%) of units that responded did not have a formal system in place for structured clinical assessment.

## Discussion

### Main findings and interpretation

This initial evaluation suggests that the use of the BSOTS increased the number of women assessed within 15 min of arrival in the triage department, and reduced the waiting time for medical review for those who required it, which may improve safety for women and babies. Consistent inter-rater reliability in the use of the tool intimates it offers a reliable method of triaging pregnant women.

The BSOTS requires that the initial triage is undertaken by one midwife and subsequent care provided by another midwife, so implementation of this system involved a major change in working practices, which can be hard to achieve [24]. However, the midwives felt the system was safer and reported they felt more in control of the Department than previously, so the majority did change their practice. As a result of exploring the views of the midwives, the validated numerical pain score was removed from the initial assessment and replaced with a clinical assessment of pain (as being none, mild, moderate or severe). Changes were also made to the documentation to eliminate repetition of information.

The system as a whole has benefits, which include ensuring standard assessments and care are undertaken. The implementation has also had unexpected positive consequences as there is now a shared understanding among staff regarding how the severity of a woman’s condition can be defined using the colour coded categories. This is beneficial for the referral and standardised handover of care of women. Use of the BSOTS also provides an overview of the workload in the department and helps ensure appropriate escalation. Some 2 years after implementation, the system is embedded in clinical practice and, thus, is likely to be improving safety for women and their babies.

Our national survey of practice demonstrates wide variation in all aspects of service provision which suggests that maternity units may be regularly experiencing midwifery red flag events related to the delay of 30 min or more between presentation and triage of women as defined by recent national guidance [25]. Implementation of BSOTS could help ensure women are seen according to their clinical priority and improve safety for women and babies. The need to develop guidelines for triage of pregnant women has been highlighted recently by the American College of Obstetricians and Gynecologists [26], which advocates the use of tools such as the one reported here to improve quality and efficiency.

### Strengths and limitations

The choice of methods to evaluate the introduction of the system was challenging. Individual randomisation of women was not feasible due to possible contamination of both women and staff, so the use of mixed methods for this initial evaluation was appropriate. The choice of primary measures was also complex. The potentially adverse events that the system is designed to prevent (critical illness of the mother or fetus/baby) are rare, so we chose to use the number of women seen within 15 min, although we could have selected the time to being seen by a doctor, for those who require it. Missing data in the medical notes, particularly regarding the timings of assessment, means these results should be treated with caution. However, we have no reason to believe that there was systematic bias in this and it is a common problem with a recent Confidential Enquiry identifying that in two thirds of the cases reviewed there were mistakes in the notes ranging from simple omissions to a complete lack of documentation of key aspects of care [27].Response rates to two of the three midwife questionnaires was 67% and 69% respectively. While we believe this is reasonable, the views of all the midwives were not captured. The response rate to the national survey was similar (63%) but response rates showed no evidence of any bias from any particular type of maternity unit.

Our sample size was based on the assumption that 60% of women were seen within 15 min of arrival before the introduction of BSOTS while in reality it was 38%- largely due to missing times not being recorded in the notes. Given the statistically significant difference the importance of this is debatable.

This paper reports the development and implementation of BSOTS in a single maternity unit and the system has been introduced in three additional diverse maternity units to further refine it and optimise successful more widespread implementation. It may be beneficial to conduct a further evaluation based on randomisation of the maternity unit (using stepped wedge or cluster trial designs for example), however having introduced the system where there was not one in place there have been perceived improvements in the safety of women and in the organisation of the department reported by the midwives and obstetricians, so there may not be equipoise, as it is hard to postulate what harm might occur as a result of use of the BSOTS.

The implementation plan and research design for this work were developed using a co-production approach [28] in order that the sustainability of the intervention was enhanced, because there is evidence to suggest that users’ participation in and ownership of an innovation increases the likelihood of its long-term success [29].

## Conclusion

The Birmingham Symptom specific Obstetric Triage System described here appears to have excellent inter- operator reliability and appears to be a reliable way of assessing the clinical priority of women. It also appears to improve the organisation of the triage department. A national survey of practice suggests a widespread need for implementation of a standardised obstetric triage system based on clinical assessment and prioritisation of need.

## Additional files

Additional file 1: Baseline characteristics of women who attended Triage (DOCX 190 kb)

Additional file 2: Diagrammatic presentation of the pathway through the triage department (DOCX 31 kb)

Additional file 3: Questionnaire for midwives immediately after training (DOCX 32 kb)

Additional file 4: 3 month Evaluation of Triage training by midwives (DOCX 21 kb)

Additional file 5: Overall midwives questionnaire (DOCX 24 kb)

## Abbreviations

BSOTS: Birmingham Symptom specific Obstetric Triage System
DG: Development Group
ICC: Intra Class Correlation
IRR: Intra Rater Reliability
MEWS: Modified Early Warning Score
MTS: Manchester Triage System
UK: United Kingdom

## References

[1] Mackway-Jones K. Emergency triage: Manchester triage group. London: BMJ Publishing Group, London, UK, 1996. ISBN: 9780727911261.

[2] Cooke MW, Jinks S (1999). Does the Manchester triage system detect the critically ill?. J Accid Emerg Med.

[3] Speake D, Teece S, Mackway-Jones K: Detecting high-risk patients with chest pain. Emerg Nurse 2003; 11: 19–21. 10.7748/en2003.09.11.5.19.c1131 Published in print: 01 September 2003. 10.7748/en2003.09.11.5.19.c1131 14533295

[4] Gerdtz MF, Collins M, Chu M (2008). Optimizing triage consistency in Australian emergency departments: the emergency triage education kit. Emerg Med Australas.

[5] Considine J, LeVasseur S, Charles A (2002). Development of physiological discriminators for the Australasian triage scale. Accid Emerg Nurs.

[6] Gerdtz MF, Chu M, Collins M (2009). Factors influencing consistency of triage using the Australasian triage scale: implications for guideline development. Emerg Med Australas.

[7] Samangaya RA, Whitworth MK, Mason J, Brockbank A, Gillham JC (2010). A maternity priority algorithm for emergency obstetric admissions. Arch Dis Child Fetal Neonatal Ed.

[8] Perry H, Lindley CM, Northover E, Connor JI, Parasuraman RA (2014). Novel system of maternity triage in the obstetric assessment unit. Arch Dis Child Fetal Neonatal Ed.

[9] Lewis G. Saving mothers’ lives: reviewing maternal deaths to make motherhood safer- 2003-2005. The seventh report on confidential enquiries into maternal deaths in the United Kingdom. 2007. CEMACH. http://www.publichealth.hscni.net/sites/default/files/Saving%20Mothers%27%20Lives%202003-05%20.pdf. ISBN: 978–0–9533536-8-2.

[10] Centre for Maternal and Child Enquiries (CMACE). Saving mothers’ lives: reviewing maternal deaths to make motherhood safer: 2006–08. The eighth report on confidential enquiries into maternal deaths in the United Kingdom. BJOG. 2011;118(Suppl. 1):1–203. doi:10.1111/j.1471-0528.2010.02847.x. 10.1111/j.1471-0528.2010.02847.x 21356004

[11] Martin S (2010). Co-production of social research: strategies for engaged scholarship. Public Money & Management.

[12] Emergency triage, second edition. Editor(s): Manchester Triage Group, Kevin Mackway-Jones, Janet Marsden, Jill Windle Blackwell Publishing Ltd,2008 ISBN: 9780470757321.

[13] The Emergency Triage Education Kit. 2007. https://health.gov.au/internet/main/publishing.nsf/Content/casemix-ED-Triage+Review+Fact+Sheet+Documents. Accessed 13 Aug 2012.

[14] Azorín J M, Cameron R. The application of mixed methods in Organisational research: a literature review. Electron J Bus Rese Methods 2010 8 (2), 95-105. Available online at www.ejbrm.com*.*

[15] Olofsson B (2013). Study Size. 2.0 ed.

[16] Cohen J. Weighted kappa: nominal scale agreement provision for scaled disagreement or partial credit. Psychol Bull. 1968;70(4):213. doi: 10.1037/h0026256 10.1037/h0026256 19673146

[17] Fleiss JL, Cohen J (1973). The equivalence of weighted kappa and the Intraclass correlation coefficient as measures of reliability. Educ Psychol Meas.

[18] Olofsson P, Gellerstedt M, Carlström ED (2009). Manchester triage in Sweden – Interrater reliability and accuracy. Int Emerg Nurs.

[19] Göransson K, Ehrenberg A, Marklund B,et al. Accuracy and concordance of nurses in emergency department triage. Scand J Caring Sci. 2005;19 (4):432-438. doi: 10.1111/j.1471-6712.2005.00372.x Article first published online: 22 NOV 2005. 10.1111/j.1471-6712.2005.00372.x 16324070

[20] Morris R, MacNeela P, Scott A (2008). Ambiguities and conflicting results: the limitations of the kappa statistic in establishing the interrater reliability of the Irish nursing minimum data set for mental health: a discussion paper. Int J Nurs Stud.

[21] Kitzinger J (1995). Introducing focus groups. Br Med J.

[22] Gale NK, Heath G, Cameron E, Rashid S, Redwood S (2013). Using the framework method for the analysis of qualitative data in multi-disciplinary health research. BioMed Central: Med Res Methodol.

[23] Cicchetti DV. Guidelines, criteria, and rules of thumb for evaluating normed and standardized assessment instruments in psychology. Psychol Assess. 1994;6(4):284. doi: org/10.1037/1040-3590.6.4.284

[24] Martin GP, Weaver S, Currie G, Finn R, McDonald R (2012). Innovation sustainability in challenging health-care contexts: embedding clinically led change in routine practice. Health Serv Manag Res.

[25] Safe midwifery staffing for maternity settings. National Institute for Health Care Excellence (NICE) Guideline NG4. Published February 2015. https://www.nice.org.uk/guidance/ng4/chapter/1-Recommendations.

[26] Hospital based triage of obstetric patients. Committee opinion no. 667. Obstet Gynecol. 2016;128:e16–9. 10.1097/AOG.0000000000001524 27333358

[27] Draper ES, Kurinczuk JJ, Kenyon S, on behalf of MBRRACE-UK (2015). MBRRACE-UK Perinatal confidential enquiry: term, singleton, normally formed, antepartum stillbirth.

[28] Hewison A, Gale N and Shapiro J. Co-production in research: some reflections on the experience of engaging practitioners in health research. Public Money & Management 2012. 32 (4), 297-302. doi: 10.1080/09540962.2012.691311. Published online: 14 May 2012.

[29] Fleiszer A R, Semenic S E, Ritchie JA, Richer M & Denis J (2015) The sustainability of healthcare innovations: a concept analysis. J Adv Nurs 71 (7), 1484–1498. doi: 10.1111/jan.12633. Article first published online: 24 FEB 2015. 10.1111/jan.12633 25708256

