# Supplementary material for: The design and implementation of an obstetric triage system for unscheduled pregnancy related attendances: a mixed methods evaluation
Source: BMC Pregnancy Childbirth. 2017 Sep 18;17:309. doi: 10.1186/s12884-017-1503-5 (PMC5604363; doi:10.1186/s12884-017-1503-5)

**Supplementary Figure 2: Diagrammatic presentation of the pathway through the triage department**


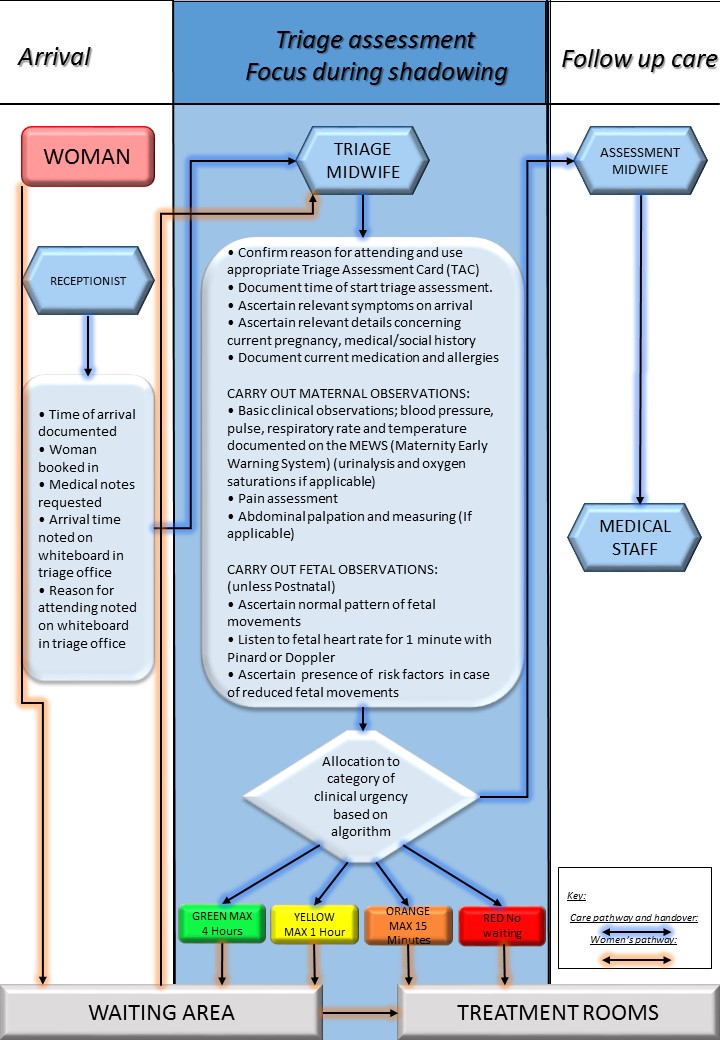

Supplement: Supplementary file 1 — Baseline characteristics of women who attended Triage (DOCX 190 kb) [file 12884_2017_1503_MOESM1_ESM.docx]
