# Supplementary material for: The design and implementation of an obstetric triage system for unscheduled pregnancy related attendances: a mixed methods evaluation
Source: BMC Pregnancy Childbirth. 2017 Sep 18;17:309. doi: 10.1186/s12884-017-1503-5 (PMC5604363; doi:10.1186/s12884-017-1503-5)
Supplement: Supplementary file 2 — Diagrammatic presentation of the pathway through the triage department (DOCX 31 kb) [file 12884_2017_1503_MOESM2_ESM.docx]

**Supplementary Table 1: Baseline characteristics of women who attended Triage**

| **Baseline Characteristics** | **Total women attended**  **n=2101** | **Women included in audit**  **n=992*** |
| --- | --- | --- |
| **Parity** |  |  |
| Nulliparous | 866 41% | 404 41% |
| Multiparous | 1137 54% | 549 55% |
| Unknown | 98 5% | 39 4% |
|  |  |  |
| **Maternal age (years)** – mean (std) | 29 (5.90) | 29 (6.01) |
|  |  |  |
| **Ethnicity** |  |  |
| Africa | 85 4% | 45 4% |
| Asia | 551 26% | 280 28% |
| Caribbean | 111 5% | 78 9% |
| European | 1014 48% | 510 51% |
| Other | 177 9% | 58 6% |
| Unknown | 163 8% | 21 2% |
|  |  |  |
| **Gestation (weeks)** |  |  |
| <20 | *Not available* | 47 5% |
| 20+1 – 28 weeks | *Not available* | 135 13% |
| 28+1 – 32 weeks | *Not available* | 95 10% |
| 32+1 – 36 weeks | *Not available* | 160 16% |
| 36+1 – 40 weeks | *Not available* | 355 36% |
| >40 weeks | *Not available* | 182 18% |
| Unknown | *Not available* | 18 2% |
|  |  |  |
| **Primary reason for attendance** |  |  |
| Abdominal pain | *Not available* | 111 11% |
| Antenatal PV bleed | *Not available* | 99 10% |
| Hypertension | *Not available* | 24 2% |
| Postnatal | *Not available* | 57 6% |
| (P)PROM | *Not available* | 116 12% |
| Reduced fetal movements | *Not available* | 190 19% |
| Suspected labour | *Not available* | 230 23% |
| Unwell/other | *Not available* | 137 14% |
| Unknown | *Not available* | 28 3% |
|  |  |  |
| **Attendance number** |  |  |
| 1 | *Not available* | 452 46% |
| 2 | *Not available* | 235 24% |
| 3 | *Not available* | 115 12% |
| 4 | *Not available* | 64 6% |
| >4 | *Not available* | 51 5% |
| Unknown | *Not available* | 75 7% |

*Data from 18 women not available and included in unknown category: 10 in 2012 and 8 in 2013

**Supplementary Table 2: Baseline characteristics of women included in audit seen in 2012 and 2013**

|  | **n=992** | |
| --- | --- | --- |
| **Baseline Characteristics** | **2012**  **n=496*** | **2013**  **n=496**** |
| **Parity** |  |  |
| Nulliparous | 198 40% | 205 41% |
| Multiparous | 276 56% | 268 54% |
| Unknown | 22 4% | 23 5% |
|  |  |  |
| **Maternal age (years)** – mean (std) | 29 (6.84) | 28 (6.11) |
|  |  |  |
| **Ethnicity** |  |  |
| Africa | 26 5% | 19 4% |
| Asia | 122 25% | 160 33% |
| Caribbean | 37 8% | 40 8% |
| European | 264 53% | 236 48% |
| Other | 31 6% | 25 5% |
| Unknown | 16 3% | 16 3% |
|  |  |  |
| **Gestation (weeks)** |  |  |
| <20 | 26 5% | 21 4% |
| 20+1 – 28 weeks | 69 14% | 66 13% |
| 28+1 – 32 weeks | 47 10% | 48 10% |
| 32+1 – 36 weeks | 76 15% | 84 17% |
| 36+1 – 40 weeks | 170 34% | 185 37% |
| >40 weeks | 98 20% | 84 17% |
| Unknown | 10 2% | 8 2% |
|  |  |  |
| **Primary reason for attendance** |  |  |
| Abdominal pain | 51 10% | 58 12% |
| Antenatal PV bleed | 54 11% | 45 9% |
| Hypertension | 10 2% | 13 2% |
| Postnatal | 22 5% | 35 6% |
| (P)PROM | 56 11% | 56 11% |
| Reduced fetal movements | 100 20% | 87 18% |
| Suspected labour | 115 23% | 111 23% |
| Unwell/other | 68 14% | 68 14% |
| Unknown | 20 4% | 23 5% |
|  |  |  |
| **Attendance number** |  |  |
| 1 | 234 47% | 213 44% |
| 2 | 109 22% | 122 25% |
| 3 | 50 10% | 62 11% |
| 4 | 28 6% | 33 6% |
| >4 | 33 7% | 18 4% |
| Unknown | 42 8% | 48 10% |

*****Notes from 10 women were not available and are included in unknown category

******Data from 8 women were not available and included in unknown category

**Supplementary Table 3: Break down by category of urgency of proportion of women assessed within 15 minutes of attendance**

| **Category of urgency** | **2012**  **n=496** | **2013**  **n=496** | **Difference**  **(95% CI)** |
| --- | --- | --- | --- |
| **Category Red *(Immediate)*** | **n=14 /421** | **n=8 /391** |  |
| Seen within 15 minutes | 11 | 4 | - |
| Overall average waiting time | 7 mins (11 mins) | 5 mins (0 mins) | -2 mins |
| Time of first assessment not available | 4 | 2 | - |
| **Category Orange *(15 mins)*** | **n=113 /421** | **n=88 /391** |  |
| Seen within 15 minutes | 39 36% | 52 59% | +23% |
| Overall average waiting time | 28 mins (37 mins) | 11 mins (22 mins) | -17 mins |
| Time of first assessment not available | 11 | 15 | - |
| **Category Yellow *(1 hour)*** | **n=223 /421** | **n=163 /391** |  |
| Seen within 15 minutes | 77 35% | 80 49% | +14% |
| Overall average waiting time | 22 mins (54 mins) | 15 mins (31 mins) | -7 mins |
| Time of first assessment not available | 20 | 30 | - |
| **Category Green *(4 hours)*** | **n=128 /421** | **n=88 /391** |  |
| Seen within 15 minutes | 32 25% | 37 42% | +17% |
| Overall average waiting time | 26 mins (40 mins) | 17 mins (32 mins) | -9 mins |
| Time of first assessment not available | 22 | 19 | - |
| **Category Unknown** | **n=18** | **n=149 /391** |  |
| Seen within 15 minutes | *not applicable* | 33 22% | - |
| Overall average waiting time | *not applicable* | 20 mins (26 mins) | - |
| Category of urgency not recorded | *not applicable* | 27 | - |
| Women not admitted to triage and missing notes | 18 | 39 | - |

**Supplementary Table 4: Comparison of time until next contact with services**

| **Attendance in Triage** | **n=992** | |
| --- | --- | --- |
|  | **2012**  **n=496*** | **2013**  **n=496**** |
| **Time until next contact** – median (std) | 8 days (9.74 days) | 5 days (8.48 days) |
| Within a day | 72 15% | 60 13% |
| Birth centre or delivery suite | 31 | 11 |
| Scheduled appointments (ANC/CMW/DAU) | 18 | 24 |
| Triage | 19 | 23 |
| Other | 3 | 1 |
| Unknown | 1 | 1 |
| Within 1 week | 133 28% | 110 24% |
| Birth centre or delivery suite | 11 | 3 |
| Scheduled appointments (ANC/CMW/DAU) | 91 | 85 |
| Triage | 26 | 21 |
| Other | 2 | 1 |
| Unknown | 3 | 0 |
| Within 2 weeks | 82 17% | 77 17% |
| Birth centre or delivery suite | 3 | 3 |
| Scheduled appointments (ANC/CMW/DAU) | 59 | 58 |
| Triage | 17 | 11 |
| Other | 3 | 3 |
| Unknown | 0 | 2 |
| Within 1 month | 43 9% | 26 6% |
| Birth centre or delivery suite | 3 | 0 |
| Scheduled appointments (ANC/CMW/DAU) | 32 | 17 |
| Triage | 5 | 5 |
| Other | 2 | 2 |
| Unknown | 1 | 2 |
| >1 month | 11 2% | 6 1% |
| Birth centre or delivery suite | 0 | 0 |
| Scheduled appointments (ANC/CMW/DAU) | 8 | 5 |
| Triage | 1 | 1 |
| Other | 2 | 0 |
| Dates Unknown | 33 7% | 34 7% |
| Birth centre or delivery suite | 12 | 5 |
| Scheduled appointments (ANC/CMW/DAU) | 2 | 2 |
| Triage | 0 | 0 |
| Other | 2 | 0 |
| Unknown | 17 | 27 |
| Not applicable (Due to Birth) | 104 21% | 144 32% |

*****Notes from 18 women were not included and presented in the unknown category for 2012: 8 were not assessed in triage and 10 sets of notes were not available.

*******Notes from 39 women were not included and presented in the unknown category for 2013: 31 were not assessed in triage and 8 sets of notes were not available.

#### Supplementary Table 5: Demographic data of midwives who undertook inter-rater reliability study

| **Demographic Questions** | | | **Entire Sample**  **(n=30)** | **Band** | |
| --- | --- | --- | --- | --- | --- |
|  |  |  | **% (n)** | **5 ^a^/6 (n=15)** | **7 (n=15)** |
| 1. | Type of staff* | a. Core midwife on DS  b. Rotational midwife | 83.3% (25)  16.7% (5) | 66.7% (10)  33.3% (5) | 100% (15)  0 (0) |
| 2. | Years worked in midwifery* | a. Less than 1 year  b. 1 – 5 years  c. 6 – 10 years  d. 11 – 15 years  e. 16 years + | 0 (0) **^b^**  20.0% (6)  33.3% (10)  6.7% (2)  40.0% (12) | 40.0% (6)  40.0% (6)  0 (0)  20.0% (3) | 0 (0)  26.7% (4)  13.3% (2)  60.0% (9) |
| 3. | How often do you work in triage | a. Daily  b. 1 – 2 times /week  c. 1 – 2 times /month  e. 1 – 2 times /3 months  d. Never | 3.3% (1)  43.3% (13)  36.7% (11)  16.7% (5)  0 (0) **^b^** | 0 (0)  26.7% (4)  46.7% (7)  26.7% (4) | 6.7% (1)  60.0% (9)  26.7% (4)  6.7% (1) |
| 4. | Highest qualification | a. Diploma  b. Graduate diploma  c. Degree  d. Masters/PhD  e. Other | 16.7% (5)  3.3% (1)  60.0% (18)  6.7% (2)  13.3% (4) | 20.0% (3)  6.7% (1)  66.7% (10)  0 (0)  6.7% (1) | 13.3% (2)  0 (0)  53.3% (8)  13.3% (2)  20.0% (3) |
| 5. | How useful is this triage system | a. Extremely useful  b. Fairly useful  c. Useful  d. Somewhat useful  e. Not at all useful | 43.3% (13)  50.0% (15)  6.7% (2)  0 (0) **^b^**  0 (0) **^b^** | 40.0% (6)  46.7% (7)  13.3% (2) | 46.7% (7)  53.3% (8)  0 (0) |
| 6. | Age range | a. 20 – 29  b. 30 – 39  c. 40 – 49  d. 50 – 59  e. 60+ | 16.7% (5)  30.0% (9)  26.7% (8)  20.0% (6)  6.7% (2) | 33.3% (5)  20.0% (3)  26.7% (4)  13.3% (2)  6.7% (1) | 0 (0)  40.0% (6)  26.7% (4)  26.7% (4)  6.7% (1) |

**^a^** Only one participant was band 5 as such they have been collapsed into the band 6 group

**^b^** Because zero, removed from subsequent analysis

* *p < .05; p <.01*  - Significant differences exist between band 6s and band 7s (fisher’s exact test utilised due to unexpected cell frequencies < 5)
