# Supplementary material for: The design and implementation of an obstetric triage system for unscheduled pregnancy related attendances: a mixed methods evaluation
Source: BMC Pregnancy Childbirth. 2017 Sep 18;17:309. doi: 10.1186/s12884-017-1503-5 (PMC5604363; doi:10.1186/s12884-017-1503-5)
Supplement: Supplementary file 3 — Questionnaire for midwives immediately after training (DOCX 32 kb) [file 12884_2017_1503_MOESM3_ESM.docx]

**Supplementary file 3**

**QUESTIONNAIRE FOR MIDWIVES**

This questionnaire will ask you some questions about the BSOTS Triage system. It would be much appreciated if you could complete the questionnaire honestly- your answers will help us decide what changes need to be made to the system.

Please answer the following questions by ticking the box or circling the answer that best applies to you:

1. **How often do you work in triage on average?**

|  | Daily |  | 1-2 times a week |  | 1-2 times a month |  | Never |
| --- | --- | --- | --- | --- | --- | --- | --- |

1. **Have you attended the BSOTS training?**

|  | Yes |  | No |
| --- | --- | --- | --- |

1. **In your view was the introduction of the new system into triage well managed?**

|  | Yes |  | No |
| --- | --- | --- | --- |

Any Comments

1. **Do you feel the BSOTS system:**
   1. **Is helpful in assessing clinical urgency accurately in women who attend Triage?**

| *Not helpful at all* | *Partly Helpful Moderately Helpful Largely Helpful* | | | *Extremely Helpful* |
| --- | --- | --- | --- | --- |
| *1* | ***2*** | ***3*** | ***4*** | ***5*** |

- 1. **Use of categories and time frames is helpful?**

| *Not helpful at all* | *Partly Helpful Moderately Helpful Largely Helpful* | | | *Extremely Helpful* |
| --- | --- | --- | --- | --- |
| *1* | ***2*** | ***3*** | ***4*** | ***5*** |

- 1. **Allows you to use your clinical judgment?**

| *Never* | *Rarely Sometimes Usually* | | | *Always* |
| --- | --- | --- | --- | --- |
| *1* | ***2*** | ***3*** | ***4*** | ***5*** |

- 1. **Enables you to accurately describe the workload in triage?**

| *Never* | *Rarely Sometimes Usually* | | | *Always* |
| --- | --- | --- | --- | --- |
| *1* | ***2*** | ***3*** | ***4*** | ***5*** |

- 1. **Enables you to obtain medical assistance more appropriately?**

| *Never* | *Rarely Sometimes Usually* | | | *Always* |
| --- | --- | --- | --- | --- |
| *1* | ***2*** | ***3*** | ***4*** | ***5*** |

1. **Means you feel more in control of the workload in Triage?**

| *Never* | *Rarely Sometimes Usually* | | | *Always* |
| --- | --- | --- | --- | --- |
| *1* | ***2*** | ***3*** | ***4*** | ***5*** |

1. **Means the Department is more organised and efficient?**

| *Never* | *Rarely Sometimes Usually* | | | *Always* |
| --- | --- | --- | --- | --- |
| *1* | ***2*** | ***3*** | ***4*** | ***5*** |

1. **Is it safer to divide the care into immediate clinical assessment and then further care and investigations by separate midwives, rather than the previous system of care by one midwife in Triage?**

|  | Agree |  | Disagree |
| --- | --- | --- | --- |

Any Comments

1. **a) Do you agree the final pain score is actually agreed between the women and the midwife?**

| *Never* | *Rarely Sometimes Usually* | | | *Always* |
| --- | --- | --- | --- | --- |
| *1* | ***2*** | ***3*** | ***4*** | ***5*** |

1. **Do you think the pain score should also include additional elements such as ability of movement and facial expression?**

|  | Yes |  | No |
| --- | --- | --- | --- |

Any Comments

1. **Regarding the paperwork for the new system, would it be helpful to combine the initial assessment and Symptom Specific Triage Assessment Card for each condition?**

|  | Yes |  | No |
| --- | --- | --- | --- |

Any Comments

1. **Would it be helpful to develop information regarding the new BSOTS system for the following?**

|  | Community Midwives |  | Local G.Ps |  | Women themselves |
| --- | --- | --- | --- | --- | --- |

Anyone else please state here

**Please answer the following questions about you**

1. **What is your age group?**

|  | 20-29 years |  | 30-39 years |  | 40-49 years |  | >50 years |
| --- | --- | --- | --- | --- | --- | --- | --- |

1. **How many years have you been working as a midwife?**

|  | <1 year |  | 1-5 years |  | 6-10 years |
| --- | --- | --- | --- | --- | --- |
|  |  |  |  |  |  |
|  | 11-15 years |  | >15 years |  |  |

1. **What is your highest qualification?**

|  | Diploma |  | Degree |  | Graduate Diploma |
| --- | --- | --- | --- | --- | --- |
|  |  |  |  |  |  |
|  | Masters/PhD |  | Registered Midwife |  |  |

1. **What band are you?**

|  | Band 5 |  | Band 6 |  | Band 7 |  | Other |
| --- | --- | --- | --- | --- | --- | --- | --- |

1. **Is there anything else you wish to tell me about triage?**

**Thank you for telling us your views!**

**Please return this to the box in triage or delivery suite once completed**
