# Supplementary material for: The design and implementation of an obstetric triage system for unscheduled pregnancy related attendances: a mixed methods evaluation
Source: BMC Pregnancy Childbirth. 2017 Sep 18;17:309. doi: 10.1186/s12884-017-1503-5 (PMC5604363; doi:10.1186/s12884-017-1503-5)
Supplement: Supplementary file 4 — 3 month Evaluation of Triage training by midwives (DOCX 21 kb) [file 12884_2017_1503_MOESM4_ESM.docx]

**Triage Training** – **Evaluation Form**

Name :

Training Date:

1. How would you rate your:
   - 1. Knowledge of what the new Triage system involves and how it will work?

|  | *Low* |  |  |  | *High* |
| --- | --- | --- | --- | --- | --- |
| *Before training* | *1* | *2* | *3* | *4* | *5* |
| *After training* | *1* | *2* | *3* | *4* | *5* |

- - 1. Confidence in assessing women and undertaking their immediate care when they attend Triage using the new system?

|  | *Low* |  |  |  | *High* |
| --- | --- | --- | --- | --- | --- |
| *Before training* | *1* | *2* | *3* | *4* | *5* |
| *After training* | *1* | *2* | *3* | *4* | *5* |

1. Was an appropriate amount of information given during the training?

| *Not enough information* | *Appropriate amount of information* | | | *Too much information* |
| --- | --- | --- | --- | --- |
| *1* | *2* | *3* | *4* | *5* |

If not enough information was given, what was missing?

1. Were any questions you had answered?

1. Can you think of any barriers to this working in practice that have not been addressed in the training?

Please write any additional comments you have here:

**Thank you!**

**Please return this once completed**
