# Supplementary material for: The design and implementation of an obstetric triage system for unscheduled pregnancy related attendances: a mixed methods evaluation
Source: BMC Pregnancy Childbirth. 2017 Sep 18;17:309. doi: 10.1186/s12884-017-1503-5 (PMC5604363; doi:10.1186/s12884-017-1503-5)
Supplement: Supplementary file 5 — Overall midwives questionnaire (DOCX 24 kb) [file 12884_2017_1503_MOESM5_ESM.docx]

**Supplementary file 4**

**3 month Evaluation of Triage training by midwives**

Name:

Today’s date:

1. Now the new Triage system have been in practice for a few months:
   - 1. Did the Triage training give you sufficient knowledge of what it involves and how it would work?

| *Low* |  |  |  | *High* |
| --- | --- | --- | --- | --- |
| *1* | *2* | *3* | *4* | *5* |

- - 1. Did the Triage training give you confidence in assessing women and undertaking their immediate care when they attend Triage?

| *Low* |  |  |  | *High* |
| --- | --- | --- | --- | --- |
| *1* | *2* | *3* | *4* | *5* |

1. Was there anything missing from the training?

|  | Yes |  | No |
| --- | --- | --- | --- |

If Yes can you tell us what?

1. Was there anything in the training that was not necessary?

|  | Yes |  | No |
| --- | --- | --- | --- |

If Yes, can you tell us what?

1. Now the new system is in practice can you tell us what is working well?

And what needs to be improved?

Please write any additional comments you have here:

**Thank you!**

**Please return this to xxx**
